# Yield improvement, grain mineral accumulation, and lipidome alteration in *Triticum durum* following *Nannochloropsis gaditana* and *Isochrysis galbana* application

**DOI:** 10.3389/fpls.2026.1868553

**Published:** 2026-07-16

**Authors:** Sana Mounaimi, Karim Lyamlouli, Ilham Zouitane, Hicham El Arroussi

**Affiliations:** 1 College of Agriculture and Environmental Sciences, AgroBioSciences Department, Mohammed VI Polytechnic University, Ben Guerir, Morocco; 2 Laboratory of Microbial Biotechnology and Bioactive Molecules, Sciences and Technology Faculty, Sidi Mohamed Ben Abdellah University, Fez, Morocco

**Keywords:** biostimulant, durum wheat, enzyme-assisted extraction, grain quality, *Nannochloropsis gaditana*, *Isochrysis galbana*, seed lipidome

## Abstract

**Introduction:**

Marine microalgae are emerging as sustainable biostimulant resources; however, species-dependent agronomic outcomes and the associated biochemical changes in cereals remain poorly understood, particularly at the seed metabolome level.

**Methods:**

In this study, we compared two metabolically distinct marine microalgae, Isochrysis galbana (Haptophyta) and Nannochloropsis gaditana (Eustigmatophyta), as enzyme-assisted extracts applied to durum wheat (*Triticum durum*) to link performance with grain composition and GC–MS seed metabolomics.

**Results:**

*I. galbana* primarily enhanced productivity, increasing grain number (+40%), grain weight (+26%), flour yield (+25%), and elevating Mn (+156%) and Ca (+14%). In contrast, *N. gaditana* promoted quality-related traits, improving ear density (+10%), flour protein (+5%), and total lipids (+177%), while showing a 3.4-fold increase in polyunsaturated fatty acids, increasing Zn (+13%), and reducing Na (–12%). Yield gains were associated with decreased thousand-kernel weight, consistent with a shift towards higher grain set with smaller kernels, while SDS sedimentation remained unchanged, indicating preserved gluten strength. Multivariate trait integration revealed distinct statistical association patterns: variables related to yield components were predominantly co-associated with *I. galbana* treatment, whereas compositional and antioxidant-related variables were predominantly co-associated with *N. gaditana* treatment. Metabolomic profiling supported these functional differences, revealing enhanced amino acid and sugar metabolism under *I. galbana* and putative lipid/redox-associated pathways under *N. gaditana*.

**Discussion:**

Collectively, these preliminary results suggest that microalgal biostimulation in durum wheat may be strongly species-specific, with seed metabolomic profiles providing integrative biochemical signatures consistent with distinct treatment effects. These observations, derived from a single growing season at one location with one cultivar, warrant multi-season and multi-location validation before species-specific biostimulant strategies can be proposed for cereal production systems.

## 1 Introduction

Feeding a growing world population while reducing the environmental footprint of agriculture represents a major challenge for contemporary agricultural systems. Wheat occupies a critical position in this context: according to FAO (2021), it ranks second for direct human consumption after rice (≈ 67 kg per capita per year) and second for feed use after maize (FAO, 2021). Wheat is cultivated at a massive scale and supports food systems worldwide, with productivity strongly influenced by agroclimatic conditions and management intensity (Shiferaw et al., 2013). Durum wheat is a major staple cereal, with global production of around 37 million tonnes per year cultivated on approximately 18 million hectares. About half of the cultivated area is concentrated in countries of the Mediterranean basin, while Canada, Mexico, and the United States are also among the leading producers (El Bzar et al., 2025). Durum wheat (*Triticum durum*) is a high-value component of this cereal landscape and a cornerstone of semolina- and pasta-based diets, where grain composition and rheological performance directly determine end-use quality.

Durum wheat is particularly sensitive to traits linked to technological quality (e.g., color attributes, hydration behavior, and dough functionality), making it an ideal model to test the capacity of biostimulants to modulate both yield and processing-related properties. Despite its caloric importance, wheat grain can be nutritionally limited, including low lysine and suboptimal concentrations of key micronutrients such as iron and zinc (Szuba-Trznadel et al., 2024). These nutritional gaps, combined with strong industrial demand for high-quality semolina, motivate approaches that enhance agronomic performance while improving grain compositional value.

Biostimulants have emerged as promising tools to support crop productivity and quality through mechanisms that go beyond direct fertilization. Microalgae, in particular, are rich in bioactive compounds (e.g., phytohormone-like molecules, amino acids, polysaccharides, antioxidants, and micronutrients) that may modulate plant metabolism and performance (Khan et al., 2018; Ronga et al., 2019). Under EU Regulation 2019/1009, plant biostimulants are framed as products that stimulate plant nutrition processes such as nutrient use efficiency, tolerance to abiotic stress, and/or quality traits without acting as fertilizers or pesticides (Jardin, 2015). While stress tolerance is a core element of many biostimulant narratives, the present study specifically targets yield- and quality-related responses under non-stress field conditions. Industry market forecasts indicate that the biostimulant sector is expanding rapidly, with estimates placing its value at over US$250 million by 2030, reflecting an annual growth rate of roughly 12.5% (Shahryari et al., 2025; Khoulati et al., 2025).

Although the beneficial effects of seaweed (macroalgae) extracts have been widely documented, the agricultural application of microalgae particularly marine species remains relatively underexplored. Seaweed-based formulations (e.g., *Ascophyllum nodosum*) dominate current markets, yet microalgal inputs offer advantages including controlled cultivation, strain-level specificity, and potentially more consistent biochemical profiles. Early work indicates that microalgae can promote plant development via improved nutrient uptake, modulation of hormone-related processes, and enhancement of antioxidant capacity (Puglisi et al., 2020; Zulfiqar et al., 2020; Mounaimi et al., 2024). However, comparatively few studies have addressed species-specific microalgal effects on cereal grain nutritional composition and metabolic pathways, especially in wheat (Ronga et al., 2019; Benedetti et al., 2018).

Among marine microalgae, *Nannochloropsis gaditana* (Eustigmatophyta) and *Isochrysis galbana* (Haptophyta) are of particular interest due to their established cultivation in aquaculture and bioindustry, their distinct biochemical signatures, and their scalability. In general, *I. galbana* is often associated with higher protein and polysaccharide fractions, whereas *N. gaditana* is noted for lipid accumulation, including polyunsaturated fatty acids and antioxidant-related metabolites (Barkia et al., 2019; Plaza et al., 2018).

Seed lipid metabolism represents a particularly relevant endpoint in cereals. Although lipids occur at lower levels than starch, they contribute to seed development, energy storage, oxidative stability, and flour/semolina functionality through components such as unsaturated fatty acids and sterols (González-Thuillier et al., 2015; Baud and Lepiniec, 2010). Moreover, lipid pathways are responsive to metabolic, hormonal, and redox signaling processes that may be modulated by biostimulant application making seed metabolomics a valuable route to connect agronomic outcomes with underlying biochemical shifts.

In this study, we investigate the species-specific potential of *Nannochloropsis gaditana* and *Isochrysis galbana* as biostimulants for *Triticum durum*, using enzyme-assisted extraction methods to evaluate their effects on growth dynamics, grain yield, and seed quality. We assess (i) their differential growth dynamics and extractability under enzyme-assisted methodologies, (ii) their effects on grain yield components and rheological behavior, and (iii) their impact on seed nutritional composition, and lipidome. Through this multidimensional approach, we aim to characterize the species-specific agronomic and biochemical responses of durum wheat to these two microalgal extracts, and to identify metabolomic signatures consistent with distinct treatment effects. The results are discussed as preliminary observations from a single field trial, intended to generate hypotheses for future mechanistic and multi-environment investigations rather than to provide operational guidelines for biostimulant deployment.

## 2 Materials and methods

### 2.1 Microalgal strains and cultivation

Marine microalgae *Isochrysis galbana* and *Nannochloropsis gaditana* (strains obtained from the Moroccan Foundation for Advanced Science, Innovation, and Research (MAScIR)) were grown in 250 mL Erlenmeyer flasks containing 200 mL of sterile F/2 medium (Guillard’s formulation), prepared with filter-sterilized seawater adjusted to pH 8.0. The medium contained NaNO_3_ (75 mg·L^−1^), NaH_2_PO_4_·2H_2_O (5.65 mg·L^−1^), Na_2_EDTA (4.16 g·L^−1^), FeCl_3_·6H_2_O (3.15 g·L^−1^), CuSO_4_·5H_2_O (10 mg·L^−1^), ZnSO_4_·7H_2_O (22 mg·L^−1^), CoCl_2_·6H_2_O (10 mg·L^−1^), MnCl_2_·4H_2_O (180 mg·L^−1^), and Na_2_MoO_4_·2H_2_O (6 mg·L^−1^). Cultures were inoculated to an initial optical density of 0.1 at 680 nm (OD_680_) and maintained on an orbital shaker at 120 rpm under continuous illumination from 60 W fluorescent lamps (≈2,500 lux).

Cells were harvested in the late exponential phase by centrifugation at 4,500 rpm for 10 min, washed twice with distilled water, and freeze-dried for subsequent analyses (Kim et al., 2012; Paramasivam et al., 2021).

### 2.2 Growth monitoring and kinetic parameters

Microalgal growth was monitored every two days for 15 days by measuring OD_680_. Growth curves were log-transformed [ln(OD)] to determine the specific growth rate (µ, day^−1^) during the exponential phase, according to:


ln(ODt)=ln(OD0)+µt

From µ, the following parameters were derived (Mutale-Joan et al., 2020):

• Doubling Time (Td):


$$Td=\;\frac{\text{ln}(2)}{µ}$$

• Generation Time (G) and Divisions per Day:

  G=Td;      Divday= 1G

• Relative Growth Rate (RGR, %):


RGR=µ ×100

• Growth Ratio (R):


$$R=\;\frac{OD\;final}{OD\;initial}$$

Where OD_final_ is the optical density at the end of the exponential or stationary phase and OD_initial_ is the optical density at the start of cultivation.

### 2.3 Characterization of the microalgae biomass

The biochemical composition of dried microalgal biomass was analyzed to determine total sugar, protein, and lipid contents.

Total sugars were quantified using the phenol–sulfuric acid method (Dubois et al., 1951). Protein content was determined by the Bradford assay (Bradford, 1976). Total lipids were extracted following the Bligh and Dyer (1959) method, adapted for aqueous biomass. One gram of dried biomass was homogenized in a water:methanol:chloroform mixture (1:1:2, v/v). The mixture was vortexed, centrifuged at 3,000 rpm for 10 min, and the lower organic phase was collected. Solvent was evaporated under nitrogen, and total lipids were quantified gravimetrically (Bligh and Dyer, 1959).

### 2.4 Microalgae extraction

Enzymatic extraction was applied to improve the extraction efficiency of bioactive compounds (Garza-Alonso et al., 2022; Habeebullah et al., 2020). Two grams of dried *I. galbana* or *N. gaditana* biomass were suspended in 100 mL distilled water and sonicated for 15 min. The suspension was adjusted to pH 5.0, supplemented with 1% (v/v) Celluclast^®^ 1.5 L (cellulase from *Trichoderma reesei*), and incubated at 50 °C for 5 h with shaking (120 rpm). After re-adjusting pH to 5.0, 1% (v/v) Viscozyme^®^ L (a multi-enzyme complex with hemicellulase activity) was added, followed by overnight incubation (16 h) at 50 °C. Extracts were centrifuged (4,500 rpm, 8 min, 4 °C), and the supernatant was stored at −20 °C for subsequent bioactivity assays. Residual biomass pellets were oven-dried at 40 °C until constant weight to calculate extraction yield.

### 2.5 Experimental setup and microalgal treatments

Field experiments were conducted during the 2021 wheat growing season (January–May) at the MAScIR experimental farm in Rabat, Morocco (34.02° N, 6.84° W; 45 m a.s.l.). The site is characterized by a Mediterranean climate, with mean temperatures ranging from approximately 12 °C in January to 22 °C in May and a long-term average annual rainfall of approximately 450 mm, concentrated primarily between November and March with a progressive drying trend from April onwards. During the 2021 growing season, rainfall distribution was above the long-term average, with timely and well-distributed precipitation from January through March supporting uniform crop establishment and vegetative development. Mean monthly temperatures during the trial period followed the typical Mediterranean seasonal pattern, with no exceptional frost or heat stress events recorded between sowing and harvest. It should be noted that the 2021 season represented a relatively favorable year for cereal production in Morocco compared to the preceding drought-affected year of 2020, and the agronomic and metabolic responses reported in this study should be interpreted within this specific seasonal context. The soil at the experimental site was classified as sandy silt and presented the following characteristics prior to sowing: pH 7.2, extractable phosphorus 42 mg·kg^−1^, total organic carbon 0.6%, total Kjeldahl nitrogen 0.07%, and organic matter content below 1%.

The field was prepared using conventional tillage practices, including plowing to a depth of 15–20 cm for seedbed preparation. A uniform mineral fertilization program was applied across all plots (identical type, timing, and rates) to standardize crop establishment conditions. The program consisted of a basal application of DAP (diammonium phosphate, 18-46-0) at 100 kg·ha^−1^ was incorporated at sowing, providing approximately 18 kg N·ha^−1^ and 46 kg P_2_O_5_·ha^−1^. Given the moderate extractable phosphorus level of the soil (42 mg·kg^−1^), no additional phosphorus top-dressing was applied. Nitrogen top-dressing was applied in two splits: ammonium nitrate (33.5% N) at tillering (Z21–29) providing 50 kg N·ha^−1^, and urea (46% N) at stem elongation (Z31–39) providing 22 kg N·ha^−1^. Fertilization type, timing, and rates were identical across all treatments and were not considered an experimental factor. To contextualize the mineral inputs delivered via the microalgal extracts relative to this basal fertilization regime, N, P, and K concentrations were measured in the stock extracts of both species prior to field application; the corresponding nutrient quantities delivered across all five application events are presented in **Table 1**. Durum wheat (*Triticum durum* Desf., cv. Karim) was sown in January 2021 at a density of 250 seeds·m^−2^, with 25 cm inter-row spacing.

**Table 1:** Elemental characterization of *Isochrysis galbana* and *Nannochloropsis gaditana* stock extracts and estimated macronutrient inputs delivered to the crop via foliar and soil drench applications, expressed as absolute quantities and as a percentage of the basal mineral fertilization dose applied uniformly across all treatments.

Parameter	Stock extract concentration (mg·L^−1^)		Total input across 5 application (g·ha^−1a^)		Basal fertilization dose (g·ha^−1b^)	Extract input as % of basal dose	
	*I. galbana*	*N. gaditana*	*I. galbana*	*N. gaditana*		*I. galbana*	*N. gaditana*
Nitrogen (N)	0.031	0.047	0.194	0.294	90,000	< 0.001%	< 0.001%
Phosphorus (P)	0.520	0.092	3.250	0.575	46,000	0.007%	0.001%
Potassium (K)	0.065	0.460	0.406	2.875	22,500	0.002%	0.013%

^a^Calculated based on application at 2% (v/v) dilution, 6.25 L·m^−2^ per application event (250 plants·m^−2^ × 25 mL·plant^−1^), across five phenological stages.

^b^Basal mineral fertilization applied uniformly across all treatments: 90 kg N·ha^−1^, 46 kg P_2_O_5_·ha^−1^ (from DAP, 100 kg·ha^−1^ at sowing), and 22.5 kg K_2_O·ha^−1^ (from NPK 15-15–15 at sowing).

The trial was established as a randomized complete block design with three treatments and three blocks. Within each block, each treatment was represented by one plot, resulting in 9 plots in total (n = 3 plots per treatment). Individual plots measured 1.5 m × 1.5 m (2.25 m^2^) and were separated by 0.5 m buffer strips to limit spray drift and cross-treatment interference. The plot was considered the experimental unit for all statistical analyses; plants within each plot were treated as subsamples.

Treatments consisted of a water-sprayed control (0% extract), a crude microalgal extract of *Isochrysis galbana* at 2% (v/v), and a crude microalgal extract of *Nannochloropsis gaditana* at 2% (v/v). Microalgal extracts were produced by enzyme-assisted extraction as described in Section 2.4, stored at −20 °C, and freshly diluted in local irrigation water prior to each application. The pH of all treatment solutions, including the control solution, was adjusted to 5.8 using 1 M NaOH or HCl to ensure consistency among treatments.

Microalgal treatments were applied using a combined foliar spray and soil drench approach to expose both aerial tissues and the root zone to the applied solutions. Each application delivered a total volume of 25 mL per plant, equally divided between foliar spraying (12.5 mL) and soil drenching at the base of the plant (12.5 mL). Based on the planting density of 250 seeds·m^−2^, this corresponded to approximately 6.25 L·m^−2^ of total solution per application event, of which 125 mL·m^−2^ consisted of microalgal extract at 2% (v/v). Applications were conducted at five defined phenological stages using the Zadoks scale: seedling establishment (Z10–13), tillering (Z21–29), stem elongation (Z31–39), heading (Z51–59), and flowering (Z61–69 (Zadoks et al., 1974). Foliar spraying was conducted under stable daytime conditions to ensure uniform coverage and minimize evaporative losses.

Irrigation was supplied uniformly to all plots using local irrigation water, following standard management practices. Any in-season nutrient supply, when applied, was delivered uniformly across all plots as fertigation or split mineral fertilization and was not considered an experimental variable.

Sampling strategy. To minimize border effects, a net plot area was defined by excluding the outer rows and approximately 25 cm from each plot edge. At physiological maturity, plants from the net plot were harvested per plot and seeds were collected. For seed biochemical analyses, seeds from the harvested net plot plants were pooled to generate one composite seed sample per plot. Composite samples (9 total; n = 3 per treatment) were used for statistical analysis, while biochemical assays were performed in technical triplicate for each plot composite. These technical triplicates were conducted for analytical precision only and were not treated as independent biological replicates in the statistical analysis. Total sugars were quantified using the phenol–sulfuric acid method (Dubois et al., 1951), soluble proteins were determined using the Bradford assay (Bradford, 1976), total lipids were extracted following the Bligh and Dyer method (Bligh and Dyer, 1959), and total polyphenols were measured using the Folin–Ciocalteu method and expressed as gallic acid equivalents (Singleton and Rossi, 1965).

### 2.6 Analysis of plant lipidomics profile using GC-MS

Lipid extraction followed a modified chloroform–methanol protocol (Mutale-Joan et al., 2020). Freeze-dried seed material (400 mg) was ground in liquid nitrogen and transferred to glass vials (Teflon-lined caps). Dodecane (10 µL, internal standard) and pre-chilled chloroform (4 mL, −20 °C) were added, vortexed (1 min), then incubated at 85 °C for 2 h and sonicated at 60 °C for 1 h. Methanol (2 mL) was subsequently added, and samples were vortexed and sonicated for 2 h. Phase separation was achieved by addition of distilled water (1 mL); the organic phase was collected and dried under nitrogen (Mutale-Joan et al., 2020).

For transesterification, dried extracts were treated with 500 µL of 6% methanolic KOH (w/v), incubated at 85 °C for 2 h and sonicated at 60 °C for 1 h (Mutale-Joan et al., 2021). After solvent evaporation, samples were reconstituted in chloroform (750 µL) and water (250 µL), vortexed, and the organic phase was recovered and stored at −20 °C until analysis. Fatty acid methyl esters (FAMEs) were analyzed by GC–MS (Agilent 7890A) using a multimode injector and a BD-ASTMD6584 column (15 m × 0.320 mm × 0.1 µm) under EI ionization Fatty acid methyl esters (FAMEs) were analyzed by GC–MS (Agilent 7890A Series, USA) using a multimode injector and a BD-ASTMD6584 column (15 m × 0.320 mm × 0.1 µm) under electron impact ionization. The extract (4 µL) was injected in 1:5 split mode using helium as carrier gas at 3 mL·min^−1^. Detection was performed in full scan mode between 30 and 1000 m/z with a gain factor of 5. Ion source and quadrupole temperatures were set at 230 °C and 150 °C, respectively. The oven temperature program was initiated at 30 °C for 1 min, then increased at 10 °C·min^−1^ to 250 °C, followed by 20 °C·min^−1^ to 340 °C. Metabolite identification was performed by spectral matching against the NIST 2017 MS Library, with a match threshold of ≥ 80% considered reliable; compounds below this threshold were classified as tentatively identified. The amount of each compound was estimated by comparison of peak areas relative to the dodecane internal standard (10 µL, added prior to extraction) to correct for run-to-run instrument variation. Formal recovery evaluation was not performed in this study, which represents a methodological limitation acknowledged in the interpretation of absolute compound abundances. Quality control was maintained through inclusion of solvent blanks between sample batches; samples showing internal standard signal variation exceeding 15% relative to the batch mean were re-injected prior to data processing (Mutale-Joan et al., 2021).

### 2.7 Assessment of technological, morphological, and elemental quality parameters in durum wheat

Grain quality evaluation included technological, physical, and chemical parameters. Protein and moisture contents were analyzed using a Foss DS 2500 NIRS system, following AACCI standard methods (39-25.01 for protein and 44-19.01 for moisture), and results were expressed on a whole-grain basis. Gluten strength was assessed indirectly through SDS-sedimentation using an SDS shaker, according to AACCI Method 56-63.01, with results expressed as sedimentation volume (mL). Milling efficiency was evaluated by flour yield (percentage of grain weight) using a Quadrumat Junior Brabander mill, following AACCI Method 26-10.02 (Johnston et al., 2019).

Dough rheology and grain quality analyses. Dough rheology was evaluated using a Brabender Farinograph, measuring water absorption (%), dough consistency (BU), development time (min), stability (min), and degree of softening (BU), according to AACCI Method 38-20.01 (Verbauwhede et al., 2018). Color measurements of both flour and whole grain were performed using a Konica Minolta colorimeter and expressed in the CIE L*a*b* system, following AACCI Method 14-22.01 (de la Hera et al., 2013). Grain morphology parameters (kernel area, perimeter, length, and width) were determined using the GrainScan imaging system (Canon Lide 220) with image analysis based on Whan et al. (2014), while thousand-kernel weight (TKW) was determined gravimetrically following ISO 520.

Elemental composition of grains was assessed by inductively coupled plasma–optical emission spectrometry (ICP-OES, Thermo Fisher ICP 7000 series) after acid digestion of finely ground seed samples. Macro- and micronutrients quantified included Zn, Fe, Mn, Mg, Ca, Na, B, Mo, and Ni (Mallick et al., 2013).

### 2.8 Statistical analysis

All data are presented as mean ± standard deviation (n = 3). Statistical significance was assessed by one-way analysis of variance (ANOVA) using OriginPro 2024 (OriginLab Corporation, USA), and mean separation was performed with Tukey’s HSD test at p < 0.05. Although the trial was established as a randomized complete block design, block effects were not explicitly incorporated into the ANOVA model due to the limited number of blocks (n = 3), which provided insufficient degrees of freedom for a reliable mixed-model variance component estimation. Spatial heterogeneity was controlled at the design stage through blocking; future trials with greater block numbers should incorporate block effects explicitly into the statistical model. For multivariate analyses, principal component analysis (PCA), hierarchical clustering heatmaps, and Pearson correlation matrices were generated in R (v4.3.2) using the packages FactoMineR, pheatmap, corrplot, and igraph, with z-score normalization applied to standardize variables. Network analyses and chord diagrams were constructed to represent metabolite–trait and trait–trait associations. All multivariate analyses, including PCA, hierarchical clustering heatmaps, Pearson correlation matrices, and network graphs, were employed as exploratory tools to identify statistical co-association patterns across treatments and traits. Given the limited number of biological replicates (n = 3 per treatment), these analyses are not intended to establish mechanistic or causal relationships. Graphical outputs were produced in R and Python for reproducibility and visualization quality.

## 3 Results

### 3.1 Growth dynamics of *I. galbana* and *N. gaditana*

**Figure 1:**
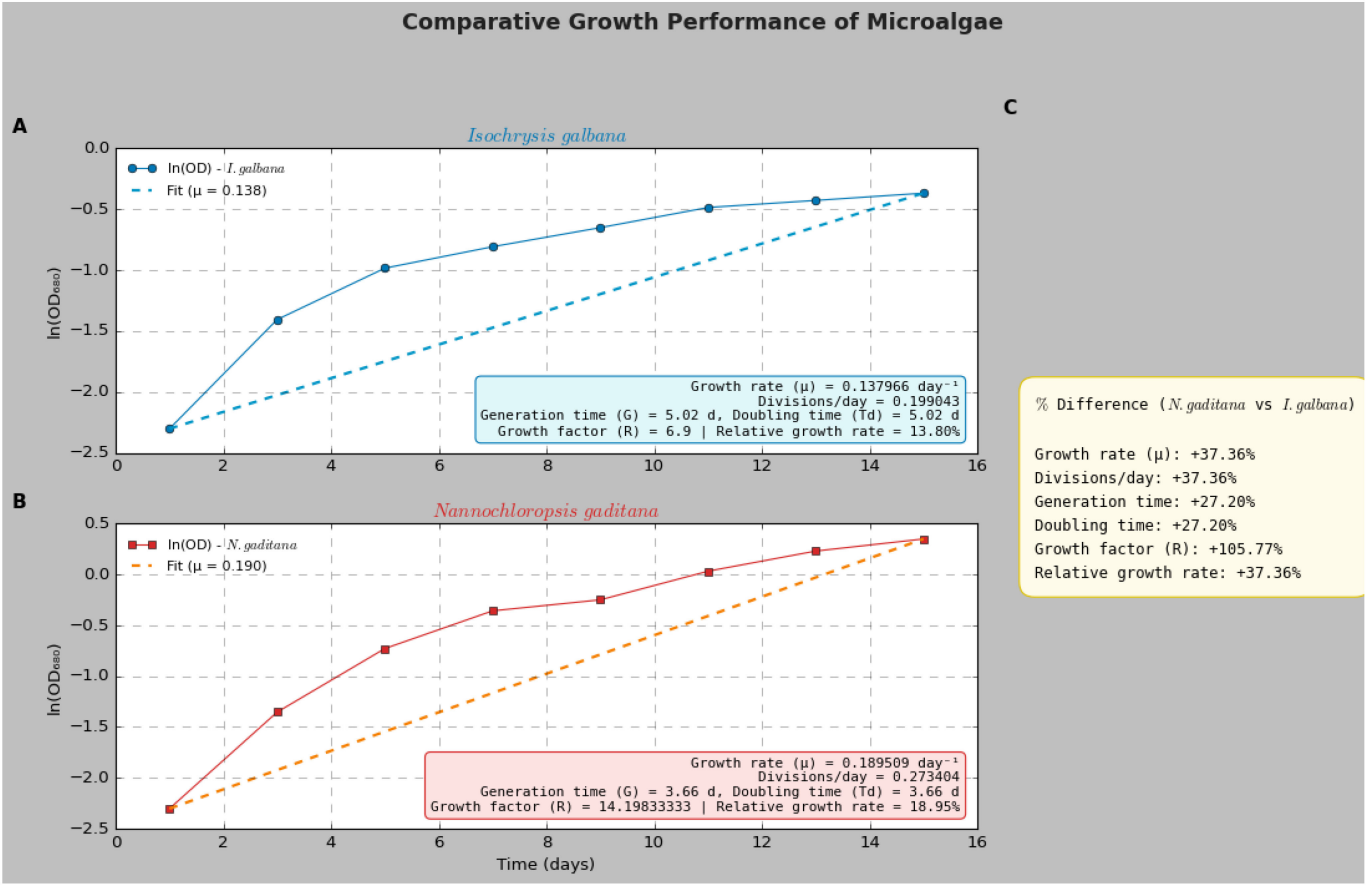
Growth of *I. galbana* and *N. gaditana* over 15 days based on OD_680_ (ln-transformed). **(A, B)** Measured values (symbols) and fitted exponential curves (dashed) for *I. galbana* and *N. gaditana*. **(C)** Percent difference of *N. gaditana* relative to *I. galbana* for key growth parameters (μ, divisions day^−1^, generation/doubling time, R, relative growth rate).

The exponential growth profiles of the two marine microalgae *I. galbana* and *N. gaditana* are shown in **Figure 1**. Optical density at 680 nm (OD_680_), expressed as the natural logarithm [ln(OD)], was monitored over 15 days to assess growth kinetics. *N. gaditana* exhibited a steeper ln(OD) slope compared with *I. galbana*, with a calculated growth rate (μ) of 0.1895 d^−1^, approximately 37.3% higher than that of *I. galbana* (μ = 0.1380 d^−1^). This indicates that *N. gaditana* grows more rapidly under the tested conditions. The exponential model provided a good fit to the observed data within the growth phase, supporting the robustness of the kinetic analysis. To aid comparison, a summary box in **Figure 1** highlights the relative percentage increase in each growth parameter for *N. gaditana* over *I. galbana*.

### 3.2 Biochemical characterization of extracts

**Figure 2:**
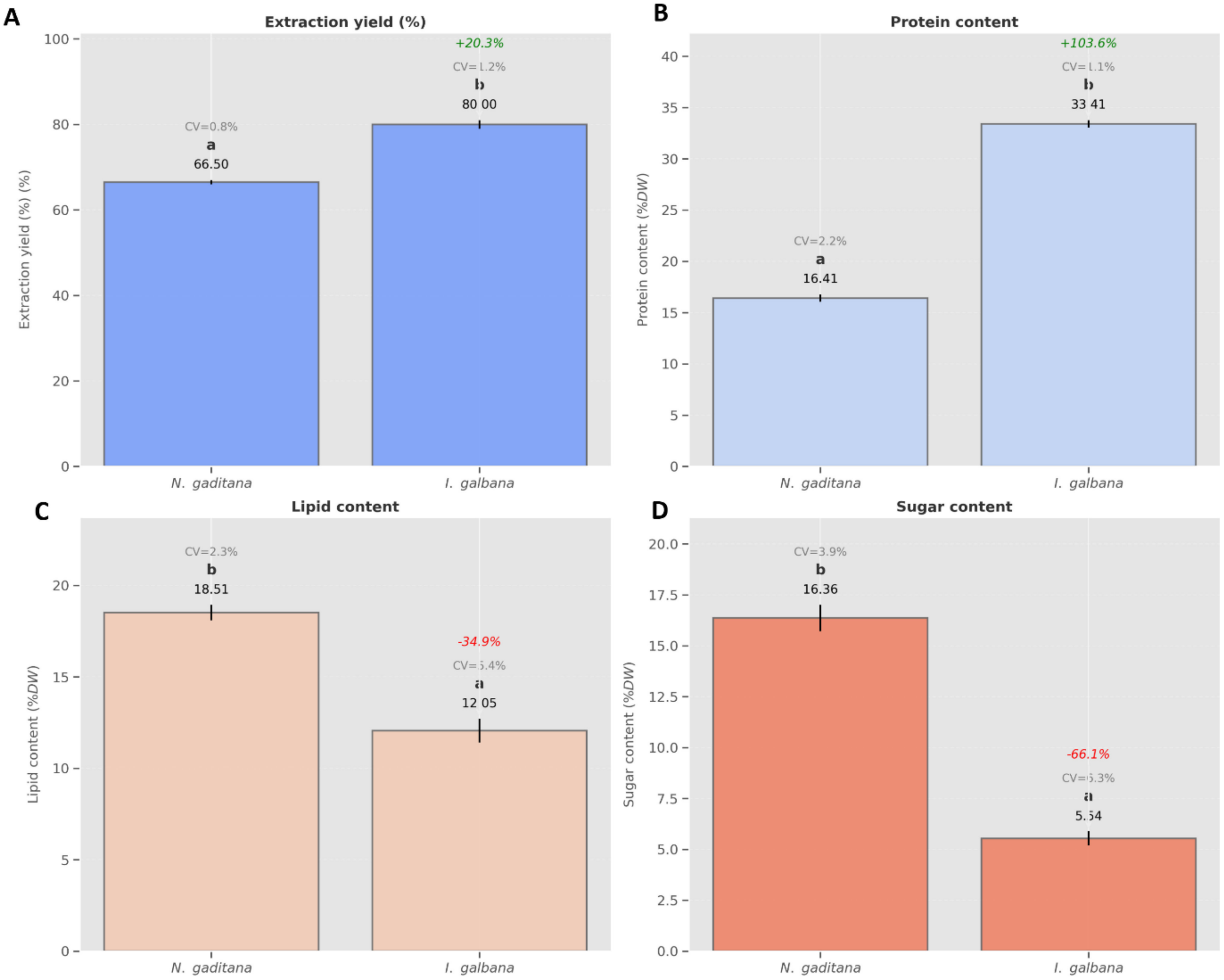
Biochemical composition of microalgal extracts from *N. gaditana* and *I. galbana*. Bars show mean ± SD (n = 3) for **(A)** extraction yield, **(B)** protein, **(C)** lipids, and **(D)** sugars (% dry weight). Percent change of *I. galbana* relative to *N. gaditana* is shown above bars. Different letters indicate significant differences (p < 0.05); CV (%) is reported for each treatment.

The biochemical profiles of *N. gaditana* and *I. galbana* extracts revealed clear compositional differences across major macromolecules (**Figure 2**).

*I. galbana* achieved a higher enzymatic extraction yield (80%) than *N. gaditana* (66.5%), reflecting greater overall recovery. Protein content was significantly higher in *I. galbana*, reaching 33.41% DW, compared to 16.41% DW in *N. gaditana*. Protein levels were highly consistent (CV = 1.1%), indicating stable performance across replicates.

In contrast, lipid accumulation favored *N. gaditana*, which recorded 18.51% DW versus 12.05% DW in *I. galbana*. Despite the higher lipid levels, *N. gaditana* showed slightly lower variability (CV = 2.3%) relative to *I. galbana* (CV = 5.4%). Sugar content showed the strongest contrast. *N. gaditana* contained 16.36% DW, while *I. galbana* had 5.54% DW.

### 3.3 Yield component response of *Triticum durum* to microalgal treatments

**Figure 3:**
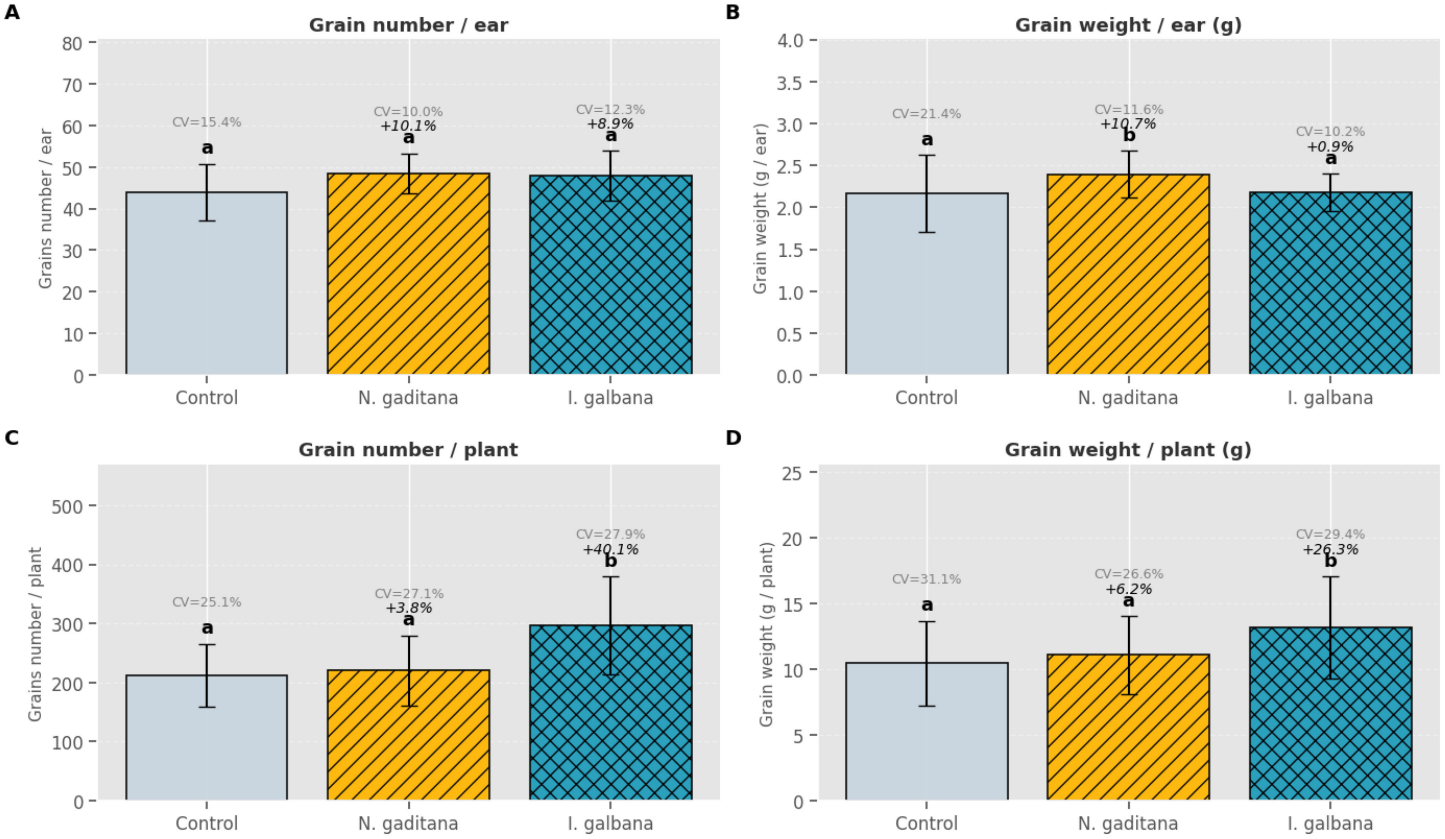
Effect of microalgal biostimulants on yield components of *Triticum durum*. Bars show mean ± SD (n = 3) for **(A)** grains ear^−1^, **(B)** grain weight ear^−1^, **(C)** grains plant^−1^, and **(D)** grain weight plant^−1^. Treatments: control (water-sprayed), *N. gaditana* extract (2%), and *I. galbana* extract (2%). Different letters indicate significant differences (ANOVA + Tukey’s HSD, p < 0.05). Percent change vs. control and CV (%) are shown above bars.

Durum wheat plants exhibited distinct responses in yield parameters when treated with microalgal extracts (**Figure 3**). Both *N. gaditana* and *I. galbana* treatments improved performance relative to the control, although the magnitude and consistency of effects differed by species and trait.

The most notable outcome was the increase in grain number per plant under *I. galbana* extract, which averaged 296.7 grains per plant, representing a 40.1% improvement over the control. This was accompanied by a 26.3% increase in grain weight per plant, indicating simultaneous gains in grain number and biomass. These effects were statistically significant (Tukey HSD, *p* < 0.05). *N. gaditana*, although showing more moderate effects overall, produced a unique 10.6% increase in grain weight per ear, surpassing *I. galbana* in this trait. Grain number per ear increased under both treatments but not significantly. Microalgal treatments also tended to reduce variability (CV%) across most yield parameters compared with the control, suggesting more consistent performance among replicates, though this observation should be interpreted cautiously given the limited number of replicates. Grain number and total grain weight per plant increased under microalgal treatments, while TKW and grain dimensions decreased, indicating a higher number of smaller grains.

### 3.4 Multidimensional effects of microalgal biostimulants on wheat grain quality

#### 3.4.1 Grain morphology and physical traits

**Figure 4:**
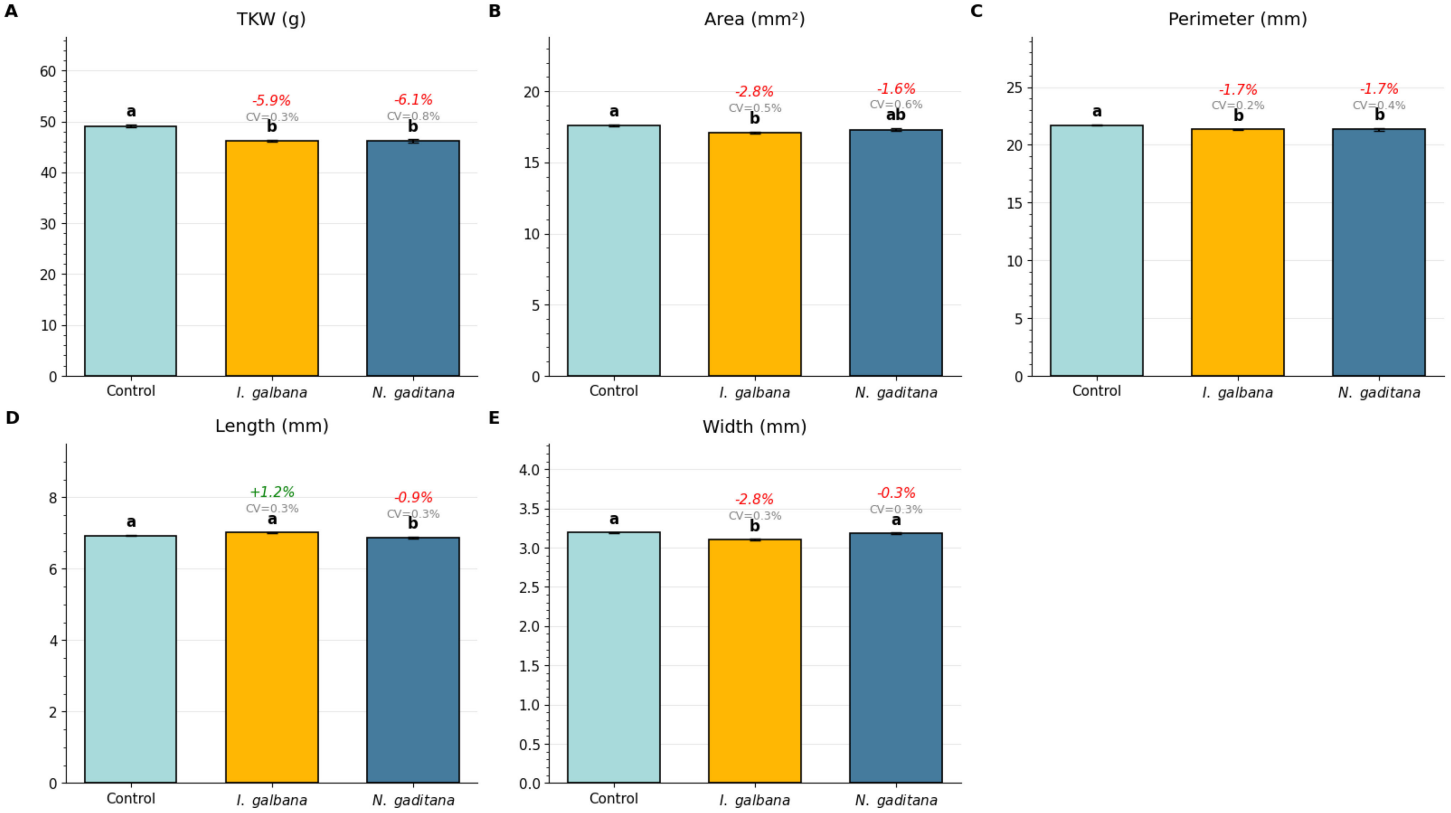
Effect of microalgal biostimulants on grain morphometric traits of *Triticum durum*. Bars show mean ± SD (n = 3) for **(A)** thousand-kernel weight (TKW), **(B)** grain area, **(C)** perimeter, **(D)** length, and **(E)** width. Treatments: control (water-sprayed), *I. galbana* extract (2%), and *N. gaditana* extract (2%). Different letters indicate significant differences (ANOVA + Tukey’s HSD, p < 0.05). Percent change vs. control and CV (%) are shown above bars.

Application of microalgal extracts led to measurable shifts in the morphological traits of durum wheat grains (**Figure 4**). Thousand kernel weight (TKW) decreased significantly by 5.9% under *I. galbana* and 6.1% under *N. gaditana* compared with the control. Grain area was also reduced (–2.8% with *I. galbana*; –1.6% with *N. gaditana*), as was grain perimeter (–1.7% in both treatments).

Grain length showed a contrasting pattern, increasing slightly (+1.2%) under *I. galbana* but decreasing (–0.9%) with *N. gaditana*. Grain width declined under *I. galbana* (–2.8%) and remained nearly unchanged under *N. gaditana* (–0.3%). Statistically significant differences were observed for TKW, area, perimeter, and width. Trait variability was low, with coefficients of variation consistently below 1.5%.

#### 3.4.2 Visual quality modulation of grains and flour

**Figure 5:**
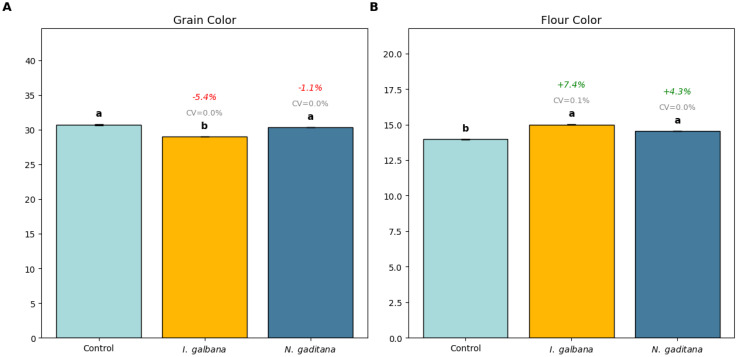
Effect of microalgal biostimulants on grain and flour color of *Triticum durum*. Bars show mean ± SD (n = 3) for **(A)** grain color and **(B)** flour color. Treatments: control (water-sprayed), *N. gaditana* extract (2%), and *I. galbana* extract (2%). Different letters indicate significant differences (ANOVA + Tukey’s HSD, p < 0.05). Percent change vs. control and CV (%) are shown above bars.

Grain and flour color were influenced by microalgal treatments (**Figure 5**). Grain brightness decreased by 5.4% under *I. galbana* and 1.1% under *N. gaditana* compared with the control, with a statistically significant reduction observed only for *I. galbana* (*p* < 0.05). In contrast, flour brightness increased markedly, by 7.4% with *I. galbana* and 4.3% with *N. gaditana*. Both treatments resulted in significantly higher flour color values than the control (*p* < 0.05), reflecting differences between whole-kernel pigmentation and the endosperm fraction measured after milling. Variability across replicates was low, with coefficients of variation consistently below 1.5%.

#### 3.4.3 Impact on processing quality traits: SDS sedimentation and moisture dynamics

**Figure 6:**
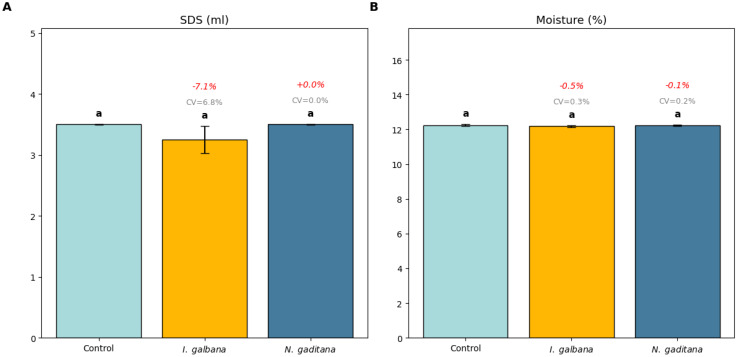
Effect of microalgal biostimulants on SDS sedimentation volume and grain moisture in *Triticum durum*. Bars show mean ± SD (n = 3) for **(A)** SDS sedimentation volume and **(B)** moisture content. Treatments: control (water-sprayed), *I. galbana* extract (2%), and *N. gaditana* extract (2%). Different letters indicate significant differences (ANOVA + Tukey’s HSD, p < 0.05). Percent change vs. control and CV (%) are shown above bars.

Processing quality was assessed through SDS sedimentation volume and seed moisture content (**Figure 6**). SDS values were unchanged under *N. gaditana* and numerically lower (–7.1%) under *I. galbana* relative to the control; however, these differences were not statistically significant (p > 0.05). Moisture content (expressed on a whole-grain basis) was consistent across all treatments, with no significant differences detected. Variation for both traits was low (CV < 2%), indicating reliable measurements.

#### 3.4.4 Seed mineral nutrition and elemental balance

**Figure 7:**
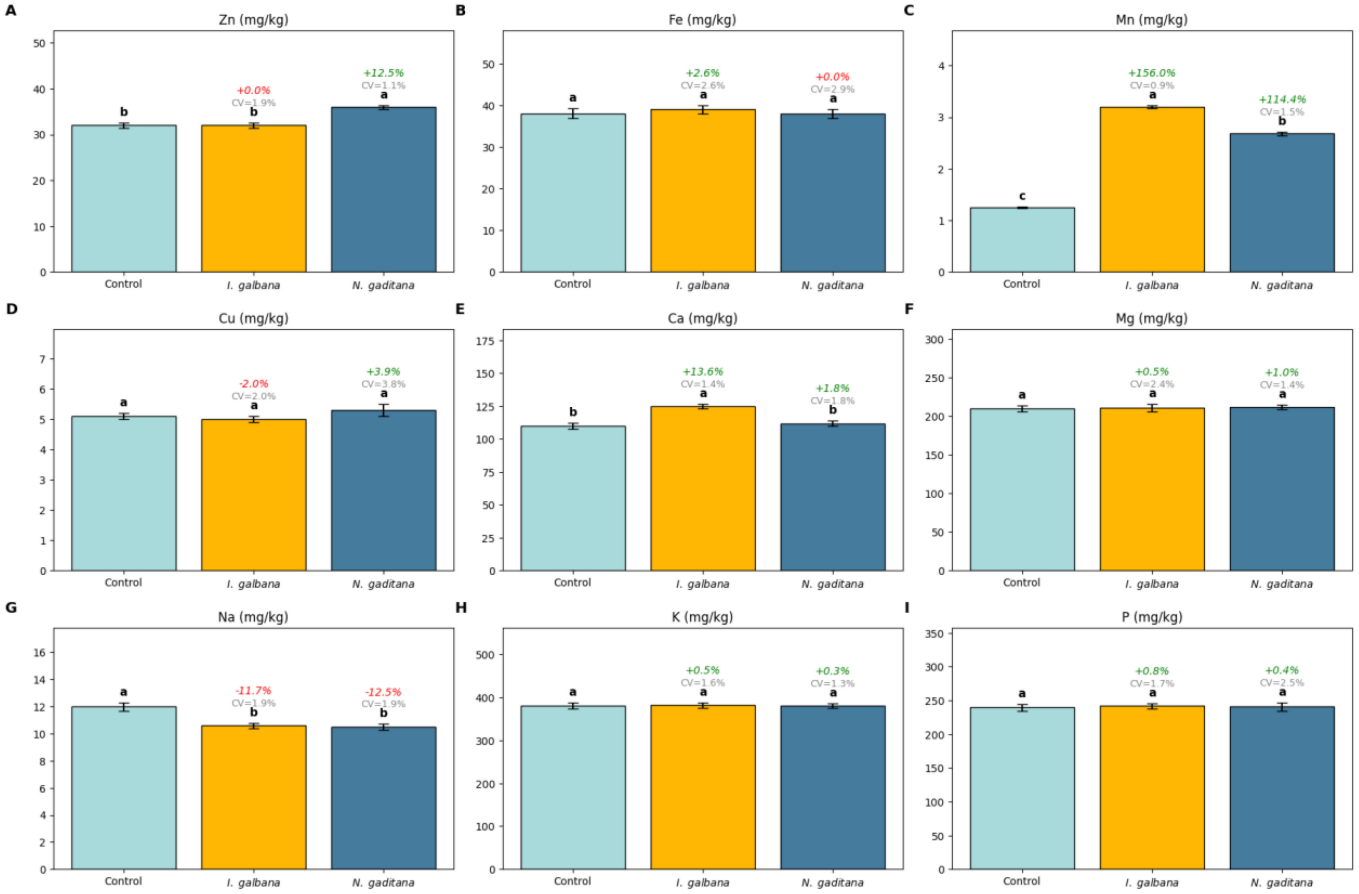
Grain mineral composition of *Triticum durum* under microalgal biostimulants. Mean ± SD (n = 3) for **(A)** Zn, **(B)** Fe, **(C)** Mn, **(D)** Cu, **(E)** Ca, **(F)** Mg, **(G)** Na, **(H)** K, and **(I)** P in control, *I. galbana* (2%), and N. gaditana (2%). Different letters indicate significant differences (ANOVA + Tukey, p < 0.05). Percent change vs. control and CV (%) are shown above bars.

Elemental analysis revealed species-specific shifts in the mineral composition of durum wheat seeds (**Figure 7**). Zinc concentration increased by 12.5% in seeds treated with *N. gaditana* (significant, p < 0.05), while no change was observed under *I. galbana*. Iron content remained stable across all treatments.

Manganese showed the strongest response, increasing by 156% with *I. galbana* and 115% with *N. gaditana*, both significantly higher than the control (p < 0.05). Calcium content also increased significantly (+13.7%) under *I. galbana*, whereas magnesium levels were unaffected.

Both treatments reduced sodium accumulation by 12% relative to the control, suggesting decreased Na uptake. Potassium and phosphorus concentrations were unchanged. Across all elements, variability was low, with CV values ranging from <1% to 8%.

#### 3.4.5 Nutritional traits of wheat seeds: protein, lipid, sugar, polyphenols responses to algal biostimulants

**Figure 8:**
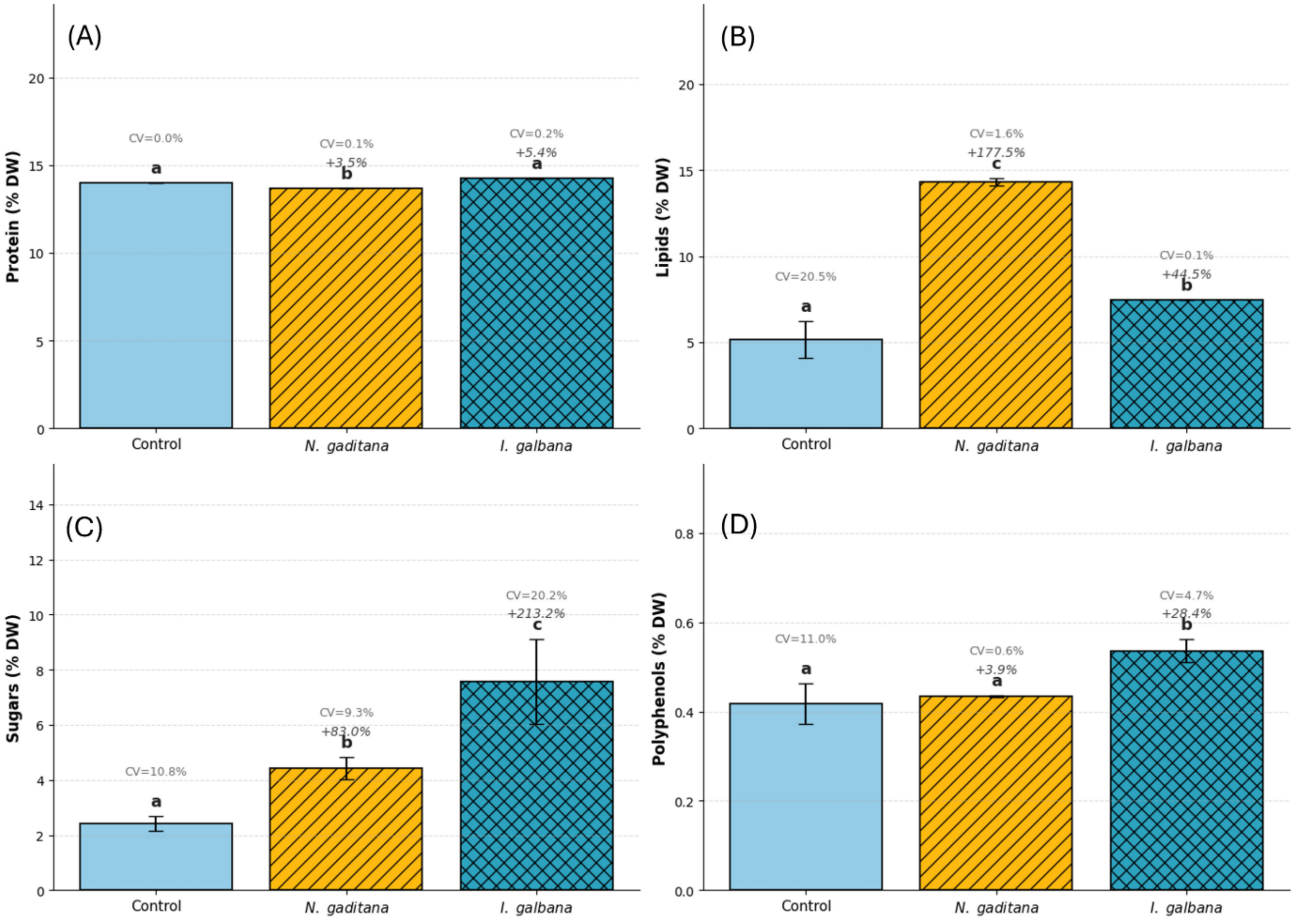
Grain biochemical composition of Triticum durum under microalgal biostimulants. Mean ± SD (n = 3) for **(A)** protein, **(B)** lipids, **(C)** sugars, and **(D)** polyphenols in control, I. galbana (2%), and N. gaditana (2%). Different letters indicate significant differences (ANOVA + Tukey, p < 0.05). Percent change vs. control and CV (%) are shown above bars.

Biochemical profiling of wheat seeds revealed distinct macronutrient responses to microalgal treatments (**Figure 8**). Grain protein content showed a numerical increase under *N. gaditana* (+5.4%) but the difference was not statistically significant (*p* > 0.05). In contrast, lipid content rose sharply, more than doubling under *N. gaditana* (+177.5%) and increasing by 44.5% under *I. galbana*, with both treatments significantly higher than the control (*p* < 0.05). Lipids also displayed the highest variability among macronutrients, with coefficients of variation up to 42%, particularly in the control condition. Sugar content increased by 213.2% in *I. galbana*-treated samples (significant, *p* < 0.05) and by 83.0% with *N. gaditana* (not significant). Overall, CV values across macronutrients ranged from <1% to 42%.

Total polyphenol content responded differently to the two treatments (**Figure 8**). *N. gaditana* induced a significant 28.4% increase compared with the control (*p* < 0.05), whereas *I. galbana* showed only a slight, non-significant increase (+3.9%). Variability was low, with coefficients of variation of 11.0% for the control, <1% under *I. galbana*, and 4.7% under *N. gaditana*.

### 3.5 GC-MS-based metabolomics of wheat seeds under microalgal treatments

The application of microalgal extracts from *I. galbana* (ISO) and *N. gaditana* (NAN) induced distinct shifts in the metabolic signature of wheat seeds, revealing treatment-specific responses across lipidic and non-lipidic compound classes. Z-score normalized heatmaps highlighted a complex reorganization of both core fatty acid metabolism and specialized pathways.

**Figure 9:**
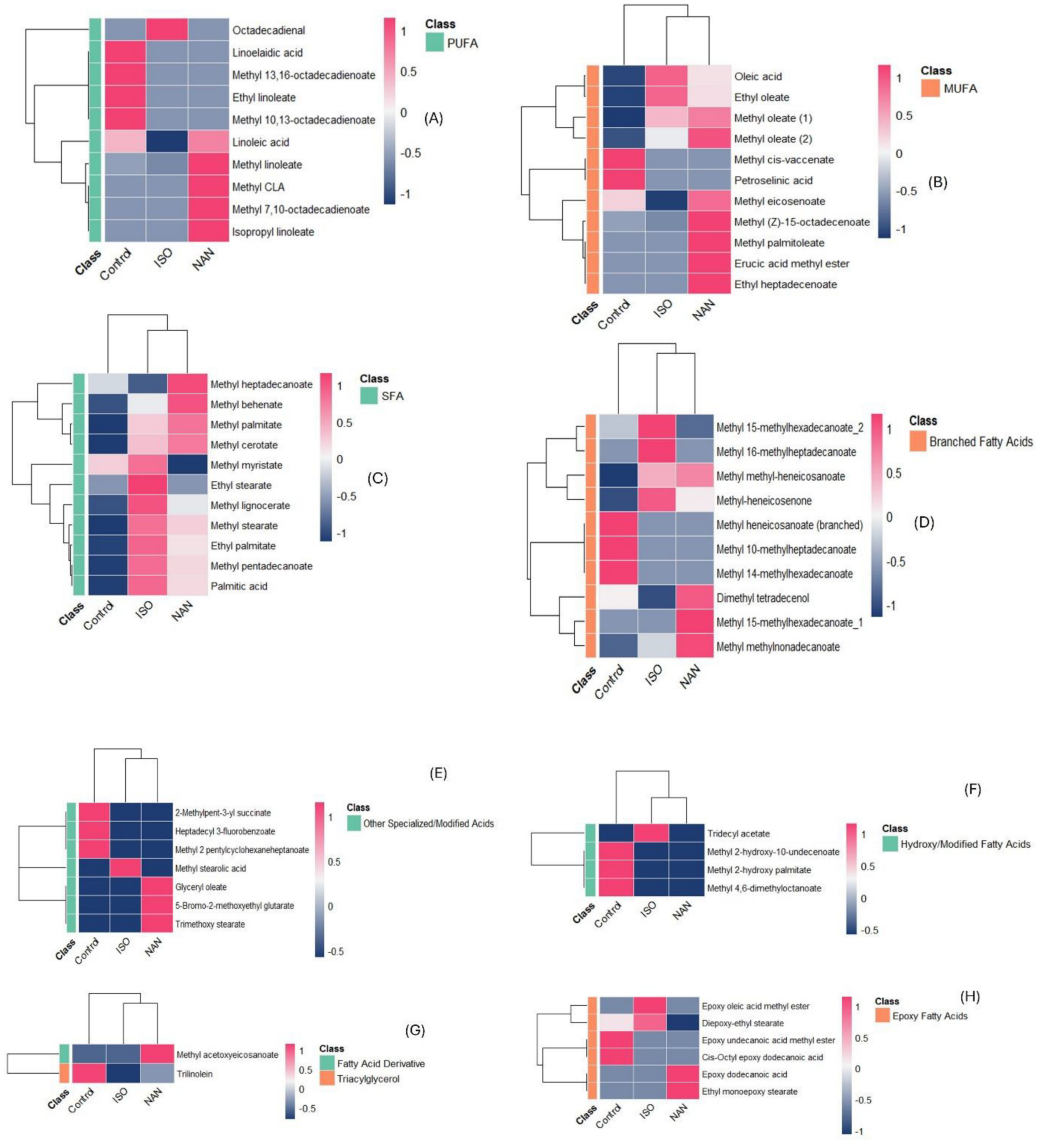
Z-score heatmap of seed fatty acids and lipid derivatives (GC–MS) in control wheat and wheat treated with *I. galbana* (ISO) or *N. gaditana* (NAN). Metabolites are grouped into eight lipid subclasses: **(A)** PUFAs, **(B)** MUFAs, **(C)** SFAs, **(D)** branched fatty acids, **(E)** other modified acids, **(F)** hydroxy/modified fatty acids, **(G)** fatty-acid derivatives & triacylglycerols, and **(H)** epoxy fatty acids. Values are row-wise Z-score–normalized (blue–red, low–high). Hierarchical clustering was applied to metabolites (rows) and treatments (columns) to identify treatment-associated co-accumulation patterns across lipid subclasses.

Within the lipidome (**Figure 9**), polyunsaturated fatty acids (PUFAs) such as methyl linoleate, linoleic acid, and conjugated linoleic acid (CLA) accumulated more strongly under NAN treatment, consistent with enhanced levels of membrane-unsaturated lipids that may contribute to stress adaptation or signaling. By contrast, ISO favored epoxy- and hydroxy-modified derivatives, including epoxy oleic acid methyl ester and methyl 2-hydroxy palmitate, consistent with oxidative lipid remodeling that may be associated with defense preconditioning, though direct physiological validation would be required to confirm this interpretation. Monounsaturated (MUFA) and saturated fatty acids (SFA) showed differential distributions, with methyl oleate and ethyl heptadecanoate enriched under ISO, while NAN promoted petroselinic acid and erucic acid derivatives. Several branched-chain fatty acids and fatty alcohols accumulated in both treatments, with a stronger signal under NAN.

**Figure 10:**
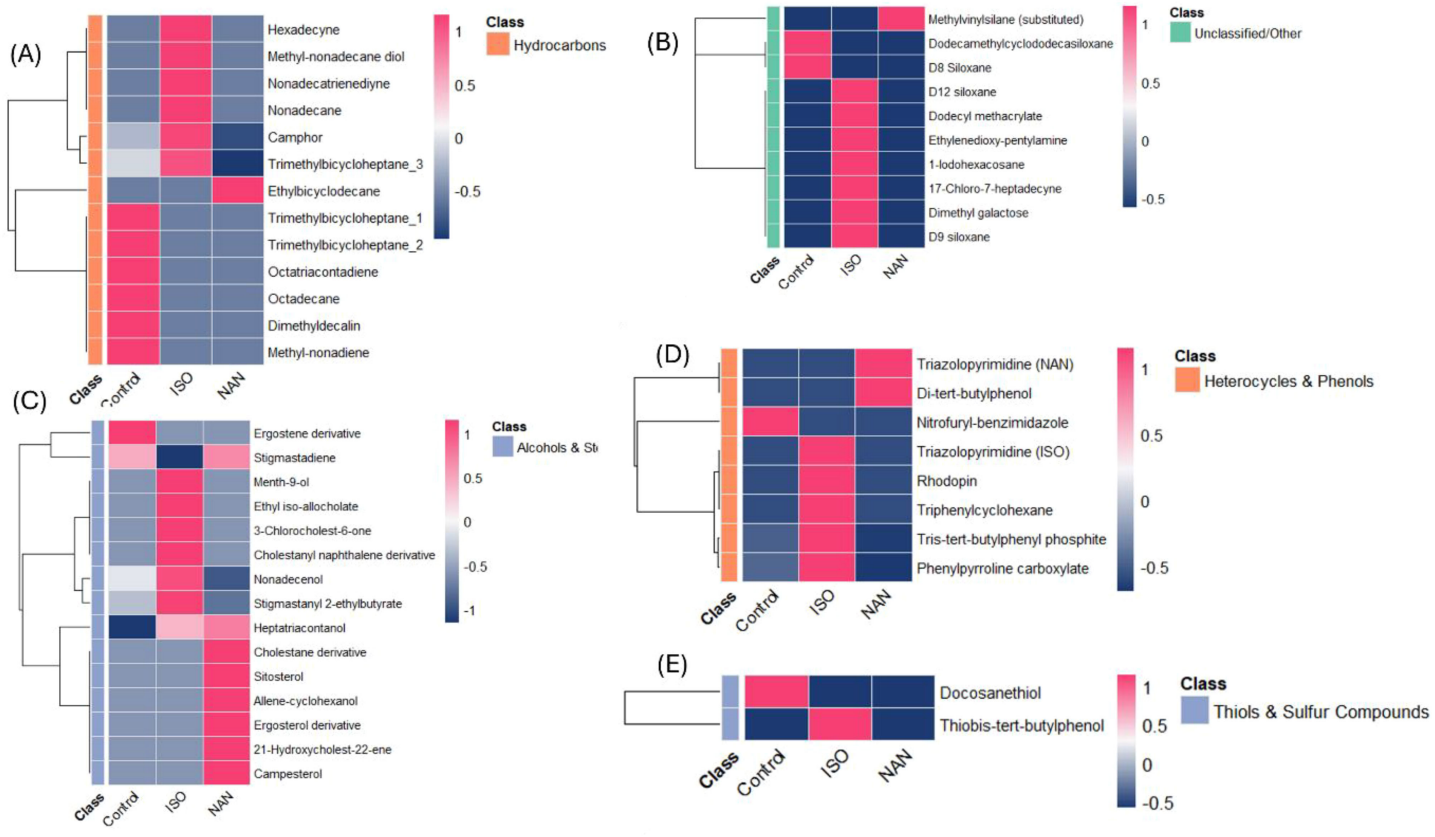
Z-score heatmap of seed hydrocarbons and related metabolite derivatives (GC–MS) in control wheat and wheat treated with (I) galbana (ISO) or (N) gaditana (NAN). Metabolites are grouped into five chemical classes: **(A)** hydrocarbons, **(B)** unclassified/other, **(C)** alcohols & sterols, **(D)** heterocycles & phenols, and **(E)** thiols & sulfur compounds. Values are row-wise Z-score–normalized (blue–red, low–high). Hierarchical clustering of metabolites (rows) reveals treatment-associated co-accumulation patterns across treatments.

Beyond fatty acids, the broader spectrum of GC–MS-detected metabolites displayed dynamic, treatment-specific patterns (**Figure 10**). Hydrocarbons, including multiple trimethylbicycloheptane isomers and long-chain alkanes, accumulated under both ISO and NAN treatments. Among the unclassified compounds, several siloxanes and methacrylate derivatives were detected at higher relative abundances in ISO-treated seeds. However, siloxanes are well-recognized analytical contaminants in GC–MS analyses, commonly arising from column bleed or sample preparation materials, and their detection should not be assigned biological significance in the absence of dedicated quality controls confirming their endogenous origin. These compounds are therefore excluded from biological interpretation in the present study. The remaining unclassified fraction, including methacrylate derivatives, is reported for completeness but treated cautiously pending further chemical characterization.

Sterols and alcohol derivatives such as nonadecenol, stigmastadiene, and ethyl iso-allocholate responded more strongly to NAN, consistent with changes in membrane-associated lipids. In contrast, carotenoid-related molecules such as rhodopin, along with triazolopyrimidine derivatives, were enriched under ISO. Sulfur-containing compounds also showed differential responses: docosanethiol was more abundant in controls, whereas thiobis-tert-butylphenol increased under ISO.

### 3.6 Effects of microalgal treatments on rheological and compositional traits of wheat flour

**Figure 11:**
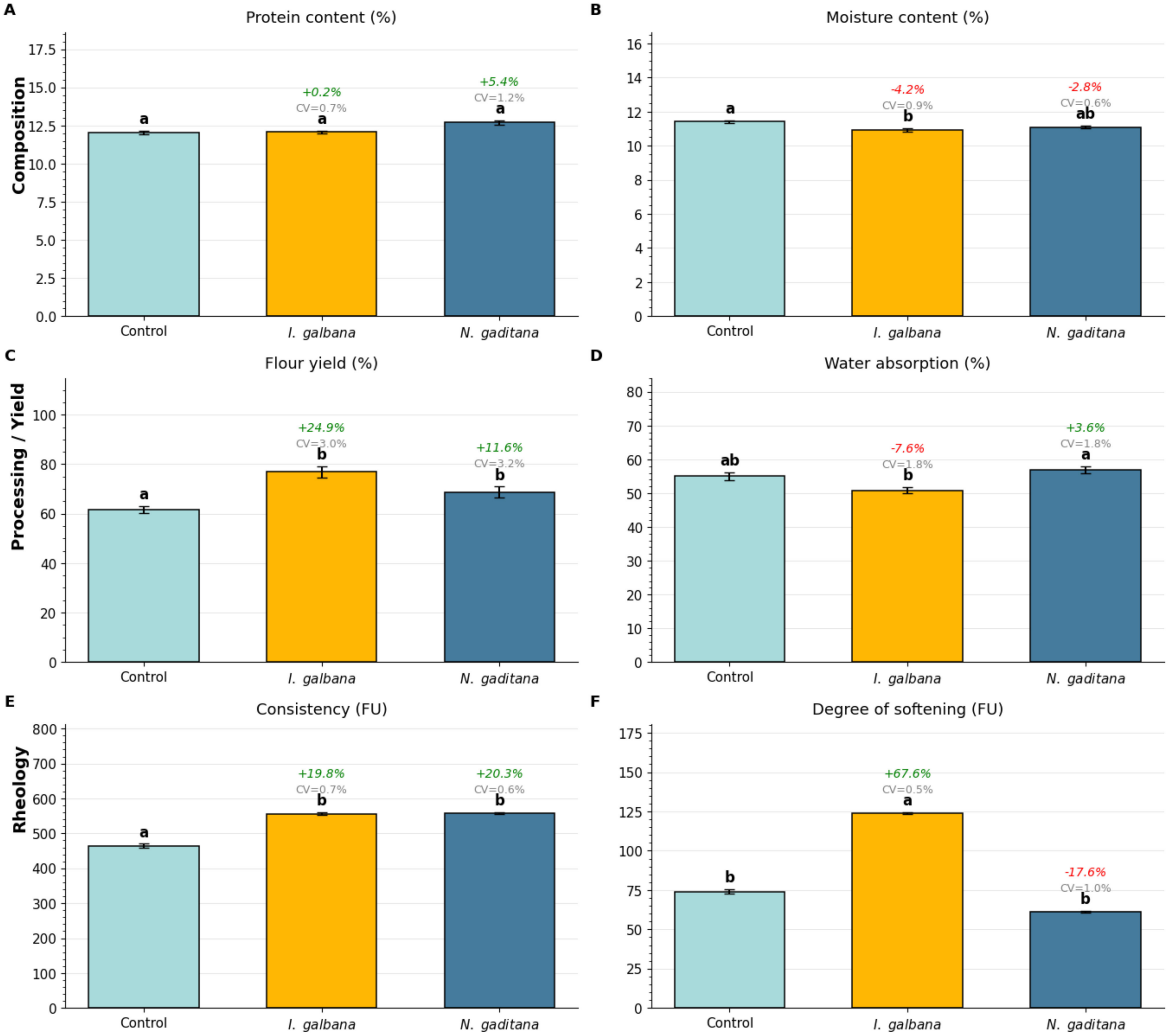
Flour composition, processing, and rheological traits of Triticum durum under microalgal biostimulants. Mean ± SD (n = 3) for **(A)** protein, **(B)** moisture, **(C)** flour yield, **(D)** water absorption, **(E)** dough consistency (FU), and **(F)** degree of softening (FU) in control, *I. galbana* (2%), and (N) gaditana (2%). Different letters indicate significant differences (ANOVA + Tukey, p < 0.05). Percent change vs. control and CV (%) are shown above bars.

Microalgal treatments affected rheological and compositional attributes of durum wheat flour (**Figure 11**). *I. galbana* increased flour yield by 24.9% compared with the control (*p* < 0.05), while *N. gaditana* increased flour yield by 11.5% (*p* < 0.05). Flour protein content was 5.4% higher under *N. gaditana* than in the control (*p* < 0.05), with no significant effect observed under *I. galbana*. Moisture content decreased slightly under *I. galbana* but remained unchanged under *N. gaditana*; these differences were not statistically significant.

The impact on dough consistency and softening dynamics was also distinct between species. Both microalgae increased dough consistency (from 464 FU to over 550 FU), with *I. galbana* and *N. gaditana* showing nearly identical enhancements (+20%). However, *I. galbana* induced a higher degree of softening (+67.6%) compared to *N. gaditana*, which slightly reduced softening values (−17.6%), pointing to contrasting effects on dough stability and strength. Finally, water absorption remained largely stable, with only minor variations across treatments. The coefficient of variation (CV%) ranged from 0.7% to 10.4%, indicating consistent data quality.

### 3.7 Multivariate patterns reveal divergent trait responses to microalgal treatments

**Figure 12:**
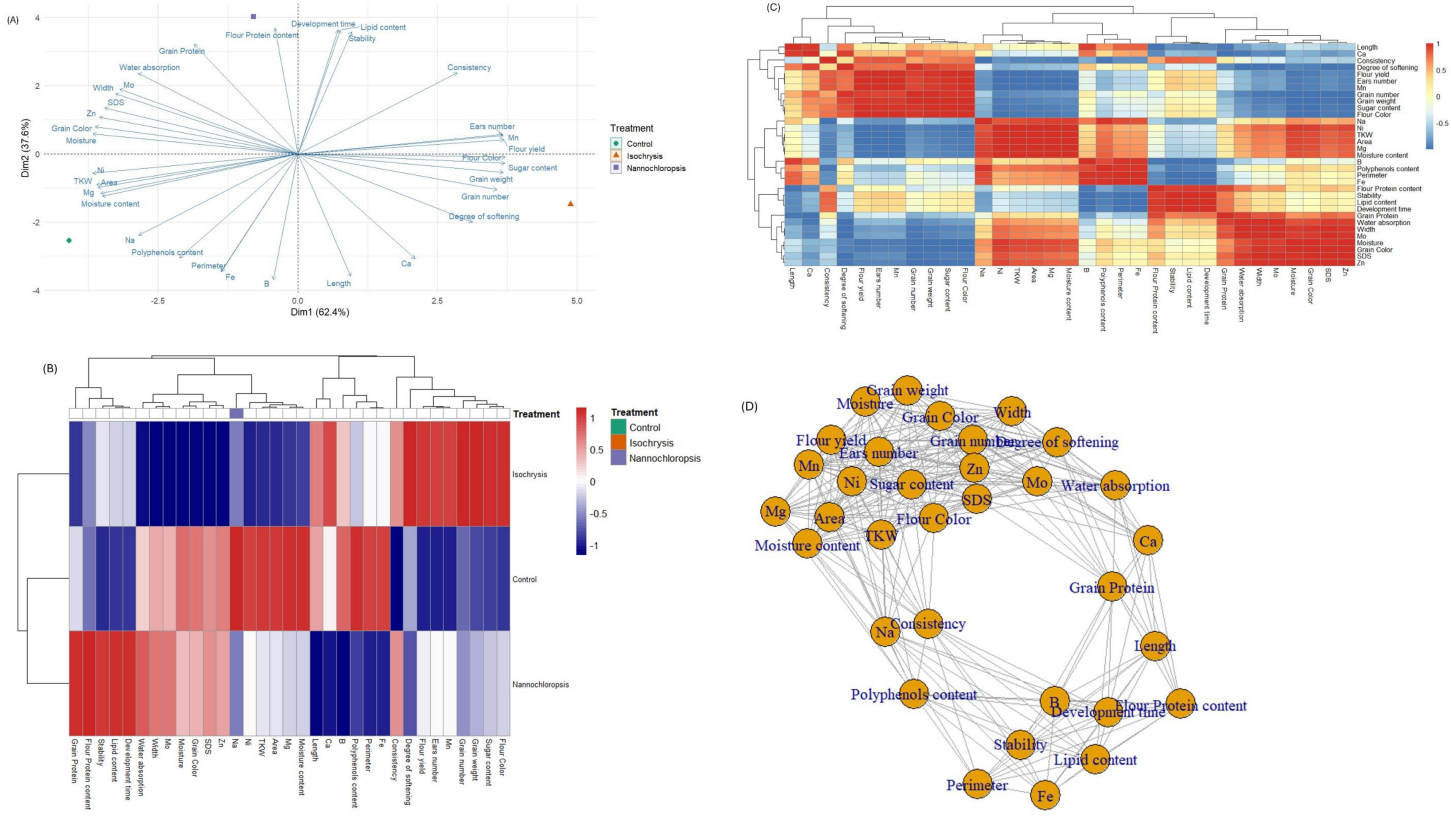
Multivariate visualization of wheat seed trait responses to microalgal treatments. **(A)** Principal Component Analysis (PCA) biplot showing treatment-associated separation and trait loadings across the first two principal components (Dim1: 62.4%, Dim2: 37.6%). Vectors represent the direction and contribution of each trait. **(B)** Scaled trait heatmap with hierarchical clustering of samples and treatments. Treatments form distinct groups with differing trait profiles. Red indicates higher scaled values, blue indicates lower values. **(C)** Pearson correlation matrix of all measured traits (ranging from morphological to compositional and rheological). Positive correlations are shown in red, negative in blue. Co-varying trait groups indicate statistical association patterns under algal treatments. **(D)** Trait network graph generated from Pearson correlations (|r| ≥ 0.7). Nodes represent traits, and edges indicate strong correlations. The network structure reveals clusters of statistically co-associated traits, with productivity traits clustering separately from biochemical and rheological traits.

Principal component analysis (PCA) revealed treatment-related differences in wheat seed traits (**Figure 12A**). The first two principal components explained 62.4% and 37.6% of the total variance, respectively. The biplot showed distinct separation between control, *I. galbana*, and *N. gaditana* groups. Vectors for grain number, grain weight, ear number, flour yield, and consistency were positioned closer to the *I. galbana* cluster. Lipid content, development time, stability, and flour protein content were aligned with the *N. gaditana* cluster, consistent with the lipid-enriched metabolomic profile reported for this treatment in section 3.5. Control samples were separated from both treatments and plotted near polyphenols, sodium, and perimeter, indicating that they contributed less to the overall variance structure despite their significant increase under *N. gaditana*.

The hierarchical clustering heatmap (**Figure 12B**). grouped samples by treatment. *I. galbana* samples clustered with higher values for sugar, flour color, and yield-related traits. *N. gaditana* samples clustered with higher values for polyphenol and lipid content. Control samples formed a separate cluster and showed comparatively lower values across most traits.

The correlation matrix (**Figure 12C**). showed strong positive associations among productivity traits, including grain weight, ear number, and flour yield. Flour quality traits such as consistency, development time, water absorption, and stability were also positively correlated. Negative correlations were observed between sodium and several structural or compositional traits. Polyphenols displayed inverse correlations with multiple growth-related traits.

To visualize these relationships more clearly, a trait network was constructed using Pearson correlation coefficients (**Figure 12D**). One cluster included lipid content, protein-related, and stability traits, while another cluster grouped productivity-related traits such as grain number, flour yield, and ear number.

Together, these multivariate approaches reveal statistical association patterns suggesting that yield-related variables were predominantly co-associated with *I. galbana* treatment, while biochemical and rheological variables were predominantly co-associated with N*. gaditana* treatment. These findings are presented as exploratory observations generated from a limited number of biological replicates and should not be interpreted as mechanistic or causal conclusions. Each treatment is nonetheless characterized by a distinct pattern of statistically co-varying traits that warrants further investigation under replicated multi-season conditions.

## 4 Discussion

### 4.1 Microalgal growth dynamics and extractability

This study presents a comprehensive and integrative evaluation of two marine microalgal biostimulants, *Isochrysis galbana (I. galbana)* and *Nannochloropsis gaditana (N. gaditana*), highlighting their distinct capacities to influence wheat productivity, biochemical traits, and grain quality through species-specific responses. By examining growth kinetics, extract composition, agronomic performance, and metabolomic profiles, our work provides a comparative perspective that may inform the selective use of microalgal extracts in strategies aimed at improving crop yield, grain mineral accumulation, and quality.

The differing growth dynamics of *I. galbana* and *N. gaditana* reveal complementary biotechnological traits with potential implications for large-scale biostimulant production. In our study, *N. gaditana* exhibited a 37.3% higher specific growth rate (μ = 0.1895 d^−1^) than *I. galbana*. This result aligns with previous reports describing high division rates and phototrophic efficiencies of *Nannochloropsis* spp. under nutrient-sufficient, light-optimized conditions (Ma et al., 2016; Paramasivam et al., 2021). These characteristics suggest that *N. gaditana* could be advantageous in biomass-oriented cultivation strategies, while *I. galbana* may offer distinct benefits in other contexts.

However, rapid proliferation did not result in a higher extractable yield. Despite its slower growth rate, *I. galbana* achieved a significantly higher extraction efficiency (+20.3%), a divergence that is worth noting given that both strains were subjected to enzyme-assisted extraction. This method, known to enhance the rupture of rigid microalgal cell walls and improve bioactive recovery, would be expected to minimize disparities caused by cell wall recalcitrance. Yet, even under enzymatic treatment, *I. galbana* displayed greater extractability, which may reflect a comparatively more accessible cell structure. Such differences could be linked to its softer cell envelope and reported higher levels of soluble exopolysaccharides, factors suggested in previous studies to support enzymatic penetration and metabolite release (Garcia-Gonzalez and Sommerfeld, 2016).

In contrast, *N. gaditana*’s algaenan-rich, multilayered cell wall may provide steric and compositional resistance to enzymatic lysis, potentially limiting the efficiency of macromolecule recovery despite elevated biomass yields. Such structural resilience, while advantageous for environmental robustness, could impose extractive challenges. Nevertheless, the fractions recovered from *N. gaditana* displayed strong bioactivity, particularly for protein- and lipid-related traits, suggesting that extraction efficiency and bioactive potency can be decoupled. Even with lower extract yield, *N. gaditana* exerted significant compositional and rheological effects in wheat, underscoring the importance of quality over quantity in biostimulant performance. These results align with the proposed concept of a biomass–extractability trade-off, whereby species optimized for volumetric productivity may nonetheless require more intensive or costly extraction strategies to achieve comparable biostimulant potency (Supraja et al., 2020).

### 4.2 Compositional profiles and implications for biostimulant function

The compositional analysis revealed clear allocation biases: *I. galbana* favored nitrogen-rich biomass (protein: 33.4% DW), while *N. gaditana* invested more in lipids (18.5% DW) and sugars (16.4% DW). These differences are consistent with reported species-specific differences in carbon allocation. Haptophytes such as *I. galbana* have been shown to accumulate proteinaceous compounds and extracellular polysaccharides under non-stressed conditions, whereas *Nannochloropsis* species are characterized by constitutive triacylglycerol accumulation, particularly when nutrients are sufficient (Ma et al., 2016; Griffiths and Harrison, 2009). The relatively high sugar content in *N. gaditana* may also reflect polyol or glucan storage, which are known to function as osmoprotectants or respiratory reserves. Notably, these distinct compositional signatures are consistent with the trait-specific effects observed in the multivariate networks. *I. galbana* appeared to be more associated with a cluster of statistically co-associated variables centered on biomass and carbohydrate allocation, whereas *N. gaditana* was linked to a cluster of variables predominantly enriched in lipid and protein traits. These associations are presented as exploratory statistical patterns rather than functionally validated modules, and their biological interpretation remains to be confirmed through experimental approaches. Such divergence supports the view that biostimulant activity may be functionally specific, with taxonomic identity determining the biochemical payload delivered to plants and potentially affecting species-dependent signaling cascades and metabolic shifts (Calvo et al., 2014; Rouphael and Colla, 2020). Although N. gaditana biomass was not protein-rich, its application was associated with a numerical but non-significant increase in wheat seed protein content (p > 0.05), which should not be interpreted as a confirmed agronomic outcome. This indicates that plant responses are not solely determined by the algal biochemical profile, but rather by the bioactivity of extractable metabolites (e.g., signaling molecules, phytohormone-like compounds) that modulate nutrient allocation in the plant.

These compositional differences have potential implications for scalable deployment, though the observations reported here derive from a single growing season, one experimental location, and one durum wheat cultivar, and should therefore be interpreted as preliminary. Subject to multi-season and multi-location validation, *N. gaditana* may be better suited to platforms where lipid-rich extracts are prioritized, provided that extraction protocols are appropriately optimized. *I. galbana* displayed higher yield-to-input ratios under enzyme-assisted extraction, suggesting potential relevance for applications requiring efficient bioactive recovery at lower biomass input. These preliminary observations suggest that strain selection for biostimulant development may benefit from considering not only growth kinetics, but also cell wall characteristics, extractability under enzymatic protocols, and the intended functional targets of the formulation factors that, in this study, appeared to differentiate the two species more meaningfully than biomass productivity alone. Integrating these dimensions may help generate more targeted hypotheses for future strain comparison studies, though definitive conclusions await broader experimental validation across seasons, locations, and extraction systems.

### 4.3 Agronomic performance: yield components and source–sink trade-offs

Both microalgal extracts enhanced grain yield components, yet their effects diverged markedly in both magnitude and physiological targets. *I. galbana* significantly increased grain number per plant (+40.1%) and total grain biomass (+26.3%), whereas *N. gaditana* selectively improved grain weight per ear (+10.6%). At the same time, both treatments reduced thousand kernel weight and grain size traits, indicating that total yield gains were achieved through a greater number of smaller grains. This contrasting response between yield components and grain morphology is consistent with a reallocation of assimilates toward grain number rather than individual grain enlargement, a trade-off commonly observed in cereals under altered source–sink balance. This divergence may reflect distinct temporal modes of action. The enhanced grain number under *I. galbana* is consistent with reports that algal bioactives can influence early developmental processes such as tillering, spikelet initiation, or floral fertility, possibly through auxin- and cytokinin-like activities described in previous studies (W. Khan et al., 2009; Stirk et al., 2014). By contrast, the increase in grain weight per ear under *N. gaditana* could be associated with later developmental processes, including assimilate loading, storage lipid deposition, or ABA-linked maturation, mechanisms that have been linked to microalgal metabolites such as PUFAs, sterols, and sugars in the literature (Guihéneuf and Stengel, 2013; Maadane et al., 2015; Martínez et al., 2022).

Moreover, the observed reduction in trait variability (CV%) under both treatments may indicate improved physiological homeostasis and stress-buffering capacity, traits that are often associated with yield stability under variable field conditions (Rouphael and Colla, 2020). The fact that both extracts were applied at identical concentrations (2%), yet elicited distinct trait-specific responses warrants attention. This observation is consistent with the hypothesis that biochemical composition may be a more decisive determinant of biostimulant response than application dose under the conditions tested, though this inference is based on a single dose level and a two-treatment comparison in one season. A dedicated dose–response experiment across multiple concentrations and environments would be required to substantiate this hypothesis.

In contrast, *N. gaditana* treatment was associated with a more pronounced increase in biochemical and rheological traits, particularly lipid content, development time, flour protein stability, and polyphenols. Although *I. galbana* displayed higher intrinsic protein content in its biomass, a numerical increase in grain seed protein was observed under though this difference did not reach statistical significance. Although polyphenols increased significantly under *N. gaditana*, their contribution to multivariate variance was smaller compared with lipid and sugar traits, explaining why they clustered with control samples in PCA and heatmap analyses. This highlights that extract composition does not necessarily translate linearly into crop protein outcomes, and suggests species-specific differences in how plant metabolism responds to distinct biochemical profiles. These outcomes are consistent with the reported metabolic profile of *Nannochloropsis* species, which are typically rich in unsaturated fatty acids and antioxidant metabolites (Guihéneuf and Stengel, 2013). Previous studies have suggested that such compounds can influence seed membrane properties, protein folding, and cellular redox balance (Rouphael and Colla, 2020). The higher Mn concentration observed under *N. gaditana* could also be linked to activation of antioxidant-related enzymes, including those in the superoxide dismutase pathway (Sharma and Dietz, 2009). Taken together, these results suggest that *N. gaditana* may be more relevant for enhancing grain quality and modifying biochemical composition, rather than for maximizing bulk yield.

### 4.4 Grain micronutrient enrichment under microalgal treatments

Our findings align with earlier reports that biostimulant inputs can selectively enrich grain micronutrients. The notably high Mn increase observed under *I. galbana* (+156%) and *N. gaditana* (+115%) was higher than the moderate Mn gains previously reported with seaweed extracts in wheat and barley, where increases were typically <50% and associated with enhanced antioxidant enzyme activity and stress tolerance (Shukla et al., 2019). This suggests that microalgal extracts may trigger a more pronounced stimulation of Mn uptake and allocation. The Zn enrichment under *N. gaditana* (+12.5%) is consistent with the mineral accumulation potential emphasized by Cakmak (2008) and falls within the range of increases reported for microbial and algal treatments in cereals (5–15%) (Rouphael and Colla, 2020; Chiaiese et al., 2018). The 12% reduction in Na under both species mirrors findings with *Ascophyllum nodosum* extracts in barley (Khan et al., 2009), reinforcing the idea that biostimulants may modulate ionic homeostasis, even under non-saline conditions. In contrast, Fe, Mg, B, Mo, and Ni remained stable, which is in line with reports that biostimulant effects on the ionome are highly selective rather than global (Bulgari et al., 2019). Taken together, these comparisons suggest that microalgal biostimulants act in a trait- and element-specific manner, with Mn and Zn emerging as the most responsive targets. This specificity is consistent with the view that their value may lie in selective modulation of nutritionally and physiologically relevant micronutrients, a pattern that would distinguish biostimulant activity from more general fertilizer-based enrichment strategies, pending mechanistic validation.

### 4.5 Metabolomic signatures and pathway

To investigate the biochemical patterns associated with the phenotypic divergence observed under microalgal treatments, we constructed an integrative metabolomic association map (**Figure 10**). This map incorporates GC–MS metabolite data, compound subclassifications, and established biosynthetic routes in cereals. By visualizing treatment-associated metabolite enrichment, the analysis reveals statistical co-association patterns between metabolite classes and treatments in *Triticum durum*, which are discussed here as hypothesis-generating observations rather than confirmed mechanistic pathways.

Treatment with *I. galbana* was associated with enrichment of metabolites linked to primary carbon metabolism, glycolysis, and amino acid biosynthesis, particularly the aspartate–glutamate pathway, which has been reported to feed into both protein synthesis and signaling cascades relevant to grain filling and growth (Hildebrandt et al., 2015). This corresponded with increases in carbohydrate-derived intermediates (e.g., malate, glycerol) and nitrogen-rich metabolites, which may support the observed gains in grain number and flour productivity. Previous studies have also noted that *I. galbana* contains phytohormones (e.g., IAA, cytokinins) and extracellular polysaccharides that could influence sink strength and source-to-sink assimilate flow, potentially favoring assimilate allocation toward grain biomass and gluten formation (Stirk et al., 2014; García-González and Sommerfeld, 2016). In contrast, *N. gaditana* showed enrichment of metabolites linked to unsaturated fatty acids (including linoleic and linolenic acid), sterols (e.g., campesterol, stigmasterol), and polyphenolic compounds classes that have been associated with seed lipid accumulation, protein stabilization, and antioxidant potential (Guihéneuf and Stengel, 2013; Maadane et al., 2015). These patterns paralleled the observed increases in flour lipid concentration, development time, and protein quality indices. Notably, enrichment of glutathione- and ascorbate-related metabolites is consistent with patterns reported for antioxidant pathway activation in seed tissues, though direct enzymatic or gene expression validation would be required to confirm pathway upregulation (Sharma and Dietz, 2006). Hormonal profiling of the extracts and multi-stage plant tissue sampling at key developmental stages such as tillering and anthesis are identified as priorities for future mechanistic investigations to substantiate the interpretive hypotheses presented here.

### 4.6 Grain biochemical and rheological traits

Rheological assays highlighted these functional contrasts. *I. galbana* was associated with increases in flour yield (+24.9%) and softening capacity, outcomes that may indicate improved milling efficiency and dough extensibility attributes often considered desirable in pasta and semolina production, where finer particle size and relaxed gluten matrices can enhance processing performance (Cuomo et al., 2024; Andrade-Linares et al., 2021; Sissons, 2008). However, increased softness could also reduce structural stability in formulations requiring robust gluten networks, underscoring a potential trade-off between extensibility and strength. In contrast, *N. gaditana* treatment increased flour protein content (+5.4%) and dough firmness, which could be consistent with tighter protein–starch interactions or gluten crosslinking reported in the literature. Such changes are generally advantageous for bread-making or high-protein flour applications, where dough strength and water absorption capacity are prioritized (Peña et al., 1995). Microalgal treatments did not significantly alter SDS sedimentation, indicating no measurable effect on gluten strength under the tested conditions. The absence of significant effects on SDS sedimentation indicates no measurable impact on gluten strength under the tested conditions. Alongside differences in flour color and softening index, these patterns highlight species-specific differences in flour macrostructural traits, a pattern consistent with distinct molecular pathways in protein polymerization and starch–gluten interactions, though direct molecular validation remains to be conducted (Gharib et al., 2024). The divergence between grain and flour color likely reflects differences in measurement scale: whole-grain color is influenced by seed coat pigmentation, whereas flour brightness depends mainly on endosperm properties and particle size distribution during milling.

These contrasting rheological profiles suggest that no single biostimulant may be optimal across all applications. These preliminary rheological contrasts suggest that cultivar × biostimulant × end-use interactions may be worth investigating as a research question in future multi-cultivar studies. However, the present design one cultivar, one season, one location does not provide a sufficient basis to generalize this pattern or to propose precision-matching frameworks for practical deployment. Whether such species-specific rheological differences hold across genotypes and environments remains to be established.

Finally, our pathway-based metabolomic mapping revealed statistical co-association patterns suggesting that *I. galbana* treatment was enriched in metabolites related to carbohydrate metabolism, glycolysis, and amino acid biosynthesis, particularly the glutamate–aspartate axis, a pattern potentially consistent with, but not causally linked to, its observed effects on grain biomass. In contrast, *N. gaditana* showed enrichment of metabolites related to fatty acid elongation, sterol biosynthesis, and antioxidant metabolism pathways classes that have been associated with seed nutritional quality in previous studies (Guihéneuf and Stengel, 2013; Maadane et al., 2015). These findings suggest that biostimulants may influence plant performance by modulating metabolic patterns rather than acting solely as nutrient inputs.

### 4.7 Perspectives for application and regulation

The species-specific associations observed in this study suggest that the selection of microalgal biostimulants may benefit from alignment with targeted agronomic objectives, rather than relying on a single formulation across diverse crop systems. In this preliminary investigation, *I. galbana* was more consistently associated with yield-related traits, while *N. gaditana* showed stronger associations with compositional and quality-related parameters. These observations were obtained under a single growing season, at one experimental location, and with one durum wheat cultivar; their generalizability across environments, cultivars, and management systems remains to be established. The incorporation of metabolomic profiles and extract characteristics into formulation strategies represents a potentially useful direction for future research, pending validation under broader agronomic conditions.

Importantly, these species-specific effects are broadly consistent with findings reported for commercial benchmarks. For example, *Ascophyllum nodosum*-based products are often associated with enhanced stress tolerance and modest yield improvements. Although no direct comparisons were made in this study, the differential associations of *I. galbana* with grain biomass and of *N. gaditana* with flour composition indicate that microalgae may offer trait-specific effects when appropriately matched to crop needs. Furthermore, the metabolite shifts observed here (e.g., lipid biosynthesis, amino acid flux, redox-related pathways) appear more specific in scope compared with the broad-spectrum activity generally attributed to brown algal extracts (Khan et al., 2009; Rouphael and Colla, 2020).

From a regulatory perspective, the distinct biochemical profiles of *I. galbana* and *N. gaditana* may have implications under EU Regulation 2019/1009, which requires substantiation of biostimulant claims with mode-of-action evidence and compositional transparency. The metabolic differences reported here represent early-stage evidence that could inform future product development strategies, though regulatory substantiation would require multi-location, multi-season data, elemental characterization of the extracts, and mechanistic validation beyond the scope of the present study (Ricci et al., 2019). Factors such as bioactive complexity, strain identity, and extraction standardization represent additional challenges for formulation development and regulatory classification. Stress tolerance claims were not evaluated here and would require dedicated experimentation under controlled stress conditions.

Finally, the pathway-level differences observed in this study are consistent with the view that biostimulants may influence plant performance by modulating metabolic patterns rather than acting solely as nutrient supplements. The statistical co-associations observed between microalgal treatments and trait-associated biochemical networks from protein-related metabolites to antioxidant-linked compounds are consistent with potential applications in crop quality improvement and increased grain mineral accumulation. Future work should focus on cultivar-specific validation under different environments, assessment of extraction efficiency relative to input costs, and evaluation of regulatory requirements to better define the scope for applied use.

The present study should be interpreted in light of several methodological considerations. Conducted over a single growing season with three replicates per treatment in a randomized complete block design, the findings represent preliminary field evidence. Multi-season and multi-location validation is necessary to account for Year × Treatment interactions and to assess the generalizability of the species-specific effects reported here. Future investigations should also incorporate elemental characterization of the extracts prior to application, alongside a nutrient-equivalent mineral control, to further substantiate the biostimulant rather than fertilization nature of the observed effects. Furthermore, future mechanistic comparisons of specific bioactive fractions should include dry weight-equivalent or total organic carbon normalization to complement the whole-extract approach used here and enable more rigorous inter-species dose comparisons.

## 5 Conclusion

This study examined the metabolic profiles and trait responses of two marine microalgae, *I. galbana* and *N. gaditana*, in *Triticum durum*, highlighting their distinct yet complementary effects. *I. galbana* was associated with increases in grain number, weight, and flour productivity, alongside enrichment of metabolites linked to carbon and amino acid metabolism. By contrast, *N. gaditana* was more closely linked with changes in fatty acid- and sterol-related metabolites, accompanied by improvements in flour protein stability and compositional quality traits.

These divergent outcomes underscore the species-specific nature of algal biostimulant effects. Rather than a one-size-fits-all solution, effective application may depend on aligning microalgal species and extract characteristics with targeted agronomic objectives. Future work should validate these findings across cultivars and environments, and evaluate extraction efficiency, economic feasibility, and regulatory considerations to inform practical deployment.

Overall, this study suggests that marine microalgae can contribute to crop improvement through multiple, composition-dependent pathways, offering a complementary tool within omics-guided and integrated biostimulant strategies for sustainable agriculture (du Jardin, 2015; Yakhin et al., 2017).

## Data Availability

The raw data supporting the conclusions of this article will be made available by the authors, without undue reservation.
